# Diagnostic Value of Circulating Progranulin and Its Receptor EphA2 in Predicting the Atheroma Burden in Patients with Coronary Artery Disease

**DOI:** 10.1155/2021/6653501

**Published:** 2021-04-21

**Authors:** Dan Tian, Qing Qin, Ruiyan Liu, Zi Wang, Xiaoyu Li, Qing Xu, Qianzhou Lv

**Affiliations:** ^1^Department of Pharmacy, Zhongshan Hospital, Fudan University, Shanghai, China; ^2^Department of Cardiology, Zhongshan Hospital, Fudan University, Shanghai Institute of Cardiovascular Disease, Shanghai, China

## Abstract

**Background:**

Progranulin (PGRN) and its potential receptor Eph-receptor tyrosine kinase-type A2 (EphA2) are inflammation-related molecules that present on the atherosclerotic plaques. However, the roles of circulating PGRN and EphA2 in coronary artery disease (CAD) remain unclear.

**Objective:**

To study the clinical significance of circulating PGRN and EphA2 levels in Chinese patients undergoing coronary angiography.

**Methods:**

Levels of circulating EphA2 fragments and PGRN were examined in 201 consecutive individuals who underwent coronary angiography for suspected CAD in our center from Jan 2020 to Oct 2020. Demographic characteristics, results of biochemical and auxiliary examinations, and other relevant information were collected. The coronary atheroma burden was quantified by the Gensini score and the existence of chronic total occlusion (CTO). Univariate analysis and multivariate logistic regression analysis were used to analyze the risk factors for acute coronary syndrome (ACS). In patients with ACS and SAP, a receiver operating characteristic (ROC) curve was generated to detect the accuracy and discriminative ability of levels of EphA2 and PGRN, the Gensini score, and cardiac injury biomarkers as surrogate endpoints for CTO.

**Results:**

Circulating EphA2 levels were significantly higher in patients with ACS than in subjects with stable angina pectoris (SAP) or control subjects (*p* < 0.001). A positive linear correlation was verified between EphA2 levels and the Gensini score (*r* = 0.306, *p* < 0.001), and negative correlation was detected with the left ventricular ejection fraction (LVEF) (*r* = −0.405, *p* < 0.001). Both PGRN and EphA2 were positively associated with cardiac injury biomarkers (i.e., NT-proBNP, cTnT, and hs-CRP) (*p* < 0.05). The area under the ROC curve of PGRN and EphA2 was 0.604 and 0.686, respectively (*p* < 0.01).

**Conclusions:**

Higher circulating EphA2 and PGRN levels were detected in patients with ACS than in patients with SAP. Circulating EphA2 and PGRN levels might be diagnostic factors for predicting the atheroma burden in patients with CAD.

## 1. Introduction

Coronary artery disease (CAD) remains the leading global cause of morbidity and mortality. Acute coronary syndrome (ACS) is an acute and dangerous type of CAD and the primary manifestation of atherosclerotic progression in the coronary artery, which is closely related to endothelial dysfunction, inflammation, and coagulation [1]. Rupture of atherosclerotic plaque and persistent thrombotic vessel occlusion at the site of plaque rupture have dominated our thinking on ACS pathophysiology. Shan et al. found that an increased baseline atheroma burden independently predicts nonculprit lesion-related major adverse cardiovascular events (MACEs) in patients with ACS after a successful culprit lesion intervention [2]. Thus, early prediction of the atheroma burden in patients with CAD plays an essential role in determining the patient prognosis. Several cardiac injury biomarkers are promising markers for atheroma burden and have been reported to predict MACEs, such as N-terminal pro B-type natriuretic peptide (NT-proBNP), cardiac troponin (cTn), and high-sensitivity C-reactive protein (hs-CRP) [3, 4].

Progranulin (PGRN) is a secreted glycoprotein encoded by the GRN gene that is implicated in multiple pathological processes such as regulation of inflammation, promotion of proliferation, mediation of cell cycle progression, cell motility, and neurotropic and lysosome regulation [5, 6]. Studies have found that plasma PGRN levels are related to insulin resistance and inflammatory regulation [7, 8]. Kojima et al. first detected the expression of PGRN in atherosclerotic plaques [9]. In animal models, PGRN deficiency leads to severe atherosclerotic lesions and increased age-related myocardial hypertrophy [10, 11].

Eph-receptor tyrosine kinase-type A2 (EphA2) is also a promising cardiovascular molecule involved in the regulation of cell-cell interactions and angiogenesis [12]. According to recent studies, EphA2^(-/-)^ApoE^(-/-)^ mice show diminished atherosclerotic plaque formation and reduced proinflammatory gene expression compared to ApoE^(-/-)^ controls, which identifies an unrecognizable role of EphA2 in regulating both plaque inflammation and progression to advanced atherosclerotic lesions [12]. Additionally, EphA2 deficiency could exacerbate the myocardial injury and the progression of ischemic cardiomyopathy [13].

Interestingly, EphA2 is a functional receptor for PGRN that has been validated in vitro, and EphA2 silencing significantly prevents PGRN-mediated autoregulation [14, 15]. Moreover, PGRN degradation into granulin peptides might exacerbate inflammation in atherosclerotic values, which contributes to the progression of atherosclerosis [16]. However, the clinical roles of PGRN and EphA2 in patients with CAD remain unclear. The study sought to identify the clinical significance of EphA2 and PGRN in patients with CAD.

## 2. Materials and Methods

### 2.1. Study Population

The study recruited adult patients scheduled for coronary angiography at the Department of Cardiology in Zhongshan Hospital Fudan University between Jan 2020 and Oct 2020. Patients were included if they were between 40 and 85 y of age. The exclusion criteria were severe infectious diseases, severe liver or kidney insufficiency, and malignant diseases. All participants provided written informed consent, and the experimental scheme was approved by the Zhongshan Hospital Institutional Ethics Committee. The study was performed in accordance with the Declaration of Helsinki.

### 2.2. Sample Collection and Measurement

Preangiography blood samples (4 mL) were obtained from the right jugular vein of patients upon admission and rapidly centrifuged at 3,000 rpm for 10 min at 4°C. Finally, approximately 2 mL of supernatants was transferred to RNase-free tubes and stored at a temperature of -80°C until subsequent assays. In all patients, the levels of EphA2 and PGRN were measured using commercial high-sensitivity enzyme-linked immunosorbent assay (ELISA) kits (RayBio Norcross, GA, and R&D Systems, Minneapolis, MN, USA, respectively) according to the manufacturer's protocols. Other blood indexes, including the levels of cardiac troponin T (cTnT), NT-proBNP, creatine kinase-MB (CK-MB), and hs-CRP, were tested by the laboratory of Zhongshan Hospital Affiliated to Fudan University using an automatic biochemistry analyzer or automatic electrochemiluminescence immunoassay analyzer.

### 2.3. Groups and Assessment of the Atheroma Burden

The results were compared between the control group, the stable angina pectoris (SAP) group, and the ACS group. The control group was defined as coronary angiography showing patency or significant stenosis of the major coronary artery (including the left anterior descending artery, circumflex artery, and right coronary artery) of less than 50%. In the case of SAP, the angina symptom should have been stable for at least 6 months with normal myocardial enzyme levels at admission and ≥50% luminal narrowing in at least one major coronary artery. ACS was defined as patients with non-ST-segment elevation myocardial infarction (NSTEMI) or ST-segment elevation myocardial infarction (STEMI) and unstable angina (UA) [17]. The Gensini scores were calculated to identify the severity and complexity of coronary atherosclerotic lesions. The Gensini score equals the sum of all segment scores (each segment score is equal to the segment weighting factor multiplied by a severity score) [18]. The segment weighting factors were 0.5, 1, 1.5, 2.5, and 5.0, according to the vascular site. Severity scores were used to reflect the specific percentage of luminal stenosis by layering 32, 16, 8, 4, 2, and 1, for 100%, 99%, 90%, 75%, 50%, and 25%, respectively. Chronic total occlusion (CTO) was defined as anterior flow TIMI = 0 in the occluded segment, and the occlusion time was at least 3 months.

### 2.4. Data Collection

Demographic information, laboratory measurements, echocardiography, the coronary angiography procedure, and medications were recorded after reviewing the laboratory database through the hospital information management system. Body mass index (BMI) was calculated as weight/height^2^ (kg/m^2^). The estimated glomerular filtration rate (eGFR) (mL/min/1.73 m^2^) was calculated from the Modification of Diet in Renal Disease (MDRD) study equation: eGFR = 175∗(Scr) − 1.154∗(age) − 0.203∗(0.742 if female) [19].

### 2.5. Statistical Analysis

Continuous data are presented as the mean and standard deviation (SD) or median (interquartile ranges). The Shapiro-Wilk test was performed to evaluate normality. Differences between groups were calculated using the one-way ANOVA or the Kruskal-Wallis test. For multiple comparisons between groups, Tukey's HSD post hoc test and the Wilcoxon rank-sum test were used. Categorical variables are presented as numbers and percentages and were compared using the chi-square test or Fisher's exact test. Spearman's rank correlation coefficients were calculated to analyze the correlation between two variables. Univariate analysis and multivariate logistic regression analysis were performed to analyze the risk factors for ACS. The forward Wald method was used to analyze the risk factors. The results are presented as odds ratios (ORs) and 95% confidence intervals (95% CIs). In patients with ACS and SAP, a receiver operating characteristic (ROC) curve was generated to detect the accuracy and discriminative ability of levels of EphA2 and PGRN, the Gensini score, and cardiac injury biomarkers as surrogate endpoints for CTO. All statistics were calculated using bilateral tests; *p* < 0.05 was considered statistically significant. All statistical analyses were performed with SPSS software (IBM SPSS Statistics 22.0).

## 3. Results

### 3.1. Baseline Characteristics of Subjects in Each Group

**Figure 1:**
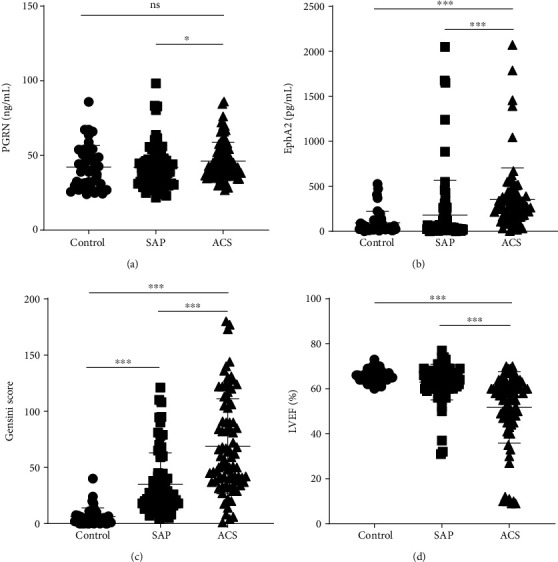
Difference of serum PGRN (a) and EphA2 (b) levels and Gensini score (c) and LVEF (d) between patients with the acute coronary syndrome (ACS) and (SAP) and control subjects.

Two hundred one patients were enrolled in the study. The baseline characteristics of the study subjects are presented in Table 1. No significant differences in demographic characteristics or risk factors were observed between groups (*p* > 0.05). However, patients with ACS showed significantly increased low-density lipoprotein (LDL) and hemoglobin A1c levels compared to control subjects (*p* < 0.05). In particular, the levels of hs-CRP, NT-proBNP, cTnT, and CK-MB were elevated in patients with ACS compared with patients with SAP or control subjects (*p* < 0.001). Circulating EphA2 levels, the Gensini score, and left ventricular ejection fraction (LVEF) were significantly higher in patients with ACS than in patients with SAP and control subjects (*p* < 0.001) (Figures 1(a), 1(c), and 1(d)). Higher PGRN levels were detected in the ACS group than in patients with SAP (*p* < 0.05) (Figure 1(b)).

### 3.2. Correlation Analysis of Circulating PGRN and EphA2 Levels

**Figure 2:**
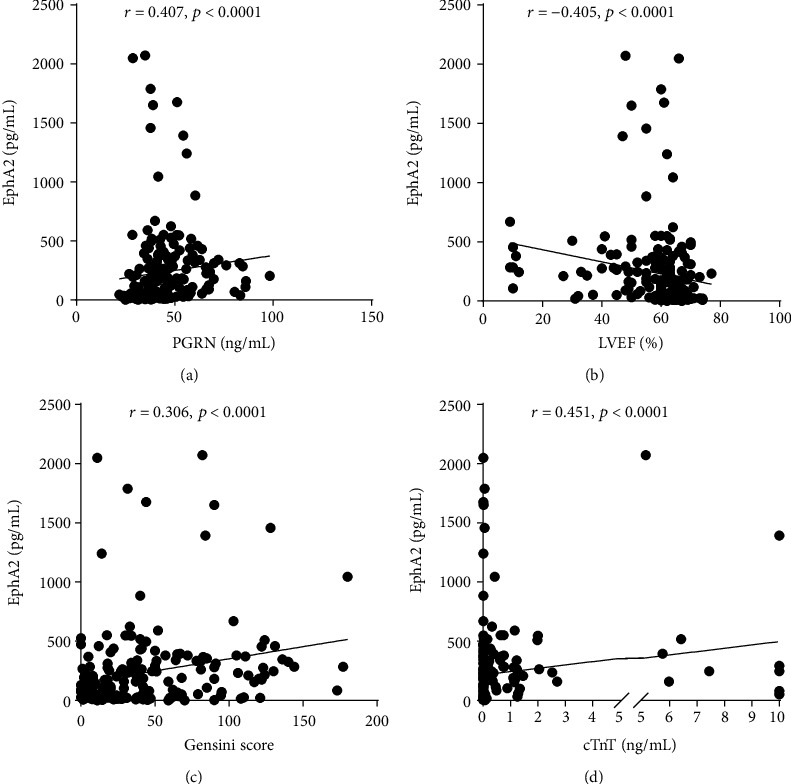
Scatterplot of correlation analysis of circulating PGRN (a), LVEF (b), Gensini score (c), and cTnT (d) with EphA2.

Spearman's correlation analysis revealed that PGRN levels had a significant positive correlation with NT-proBNP, cTnT, and hs-CRP levels (*p* < 0.05) (Table 2). EphA2 levels were correlated with total cholesterol, LDL, NT-proBNP, cTnT, CK-MB, and hs-CRP levels (*p* < 0.01). Remarkably, serum EphA2 levels were moderately positively correlated with PGRN levels (*r* = 0.407, *p* < 0.001) and the Gensini score (*r* = 0.306, *p* < 0.001) (Table 2 and Figure 2). Both PGRN and EphA2 levels were correlated with the CTO (*r* = 0.147, *p* = 0.041; *r* = 0.330, *p* < 0.001). EphA2 and PGRN levels were significantly negatively correlated with the LVEF (*r* = −0.405, *p* < 0.001; *r* = −0.215, *p* = 0.003, respectively).

### 3.3. Analysis of Risk Factors for ACS

Based on the results described above, we further explored the possibility that EphA2 and PGRN may predict ACS. The risk factors for ACS were analyzed using a univariate analysis, and risk factors with *p* < 0.05 were included in the multivariate logistic regression analysis. The male sex, LDL levels, NT-proBNP levels, EphA2 levels, and Gensini scores were associated with ACS. The OR value for males was 8.523 (95% CI: 1.422-51.091, *p* = 0.019), LDL was 1.050 (95% CI: 1.014-1.087, *p* = 0.006), NT-proBNP was 1.003 (95% CI: 1.001-1.005, *p* = 0.006), EphA2 levels ≥ 116.32 pg/mL was 10.715 (95% CI: 3.259-35.227, *p* < 0.001), and the Gensini score was 1.031 (95% CI: 1.014-1.048, *p* < 0.001) (Table 3).

### 3.4. Features of Circulating EphA2 and PGRN Levels in Patients with ACS or SAP

One hundred fifty-nine patients were diagnosed with ACS or SAP. The median Gensini score was 41.0. All patients were divided into a group with a lower atheroma burden (patients with a Gensini score < 41.0, *n* = 80) and greater atheroma burden (patients with a Gensini score ≥ 41.0, *n* = 79) (Table 4). Patients with greater atheroma burden had significantly higher EphA2 levels than patients with lower atheroma burden [243.8 (56.1, 368.8) vs. 107.8 (27.0, 273.7) pg/mL, *p* = 0.031)], whereas PGRN levels were similar between groups (*p* = 0.208). Furthermore, patients with a greater atheroma burden tended to present greater increases in total cholesterol, LDL, NT-proBNP, cTnT, CK-MB, and hs-CRP levels (*p* < 0.01). Then, patients in the CTO group (*n* = 46) and the non-CTO group (*n* = 113) were compared (Table 4). Significant differences were not observed in demographic characteristics and risk factors between groups (*p* > 0.05). Consistent with the previous result, patients with CTO had significantly higher EphA2 levels and a tendency toward elevated PGRN levels than patients without CTO [298.0 (215.5, 379.2) vs. 45.7 (23.1, 268.2), *p* < 0.001, for EphA2 and 44.7 (39.7, 51.0) vs. 39.4 (33.8, 46.7), *p* = 0.052, for PGRN]. Total cholesterol, LDL, NT-proBNP, cTnT, CK-MB, and hs-CRP levels were higher in the CTO group than in the non-CTO group (*p* < 0.05).

### 3.5. Diagnostic Efficiency of Circulating EphA2 and PGRN Levels to Predict CTO

**Figure 3:**
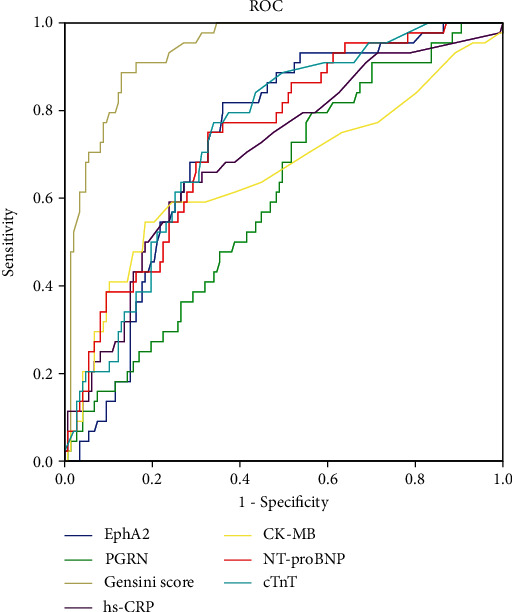
Receiver operating curve (ROC) statistics. ROC analysis demonstrates the diagnostic efficiency of seven parameters (EphA2, PGRN, Gensini score, hs-CRP, CK-MB, NT-proBNP, and cTnT) to predict CTO. CTO: chronic total occlusion.

We analyzed the diagnostic efficiency of circulating EphA2 and PGRN levels, the Gensini score, and cardiac injury biomarkers (NT-proBNP, cTnT, CK-MB, and hs-CRP) in predicting CTO in patients with ACS or SAP. As shown in Figure 3, the Gensini score showed a good discriminative ability to predict the presence of CTO (AUC of 0.911, 95% CI 0.864-0.958). Levels of EphA2, PGRN, and cardiac injury biomarkers significantly predicted the presence of CTO with low to moderate diagnostic efficiency (AUC > 0.6) (Figure 3 and Table 5). The optimal cutoff for EphA2 was 63.6 pg/mL (sensitivity at the optimal cutoff of 89.1% and specificity at the optimal cutoff of 46.3%). Moreover, the optimal cutoff for PGRN was 38.2 ng/mL (sensitivity at the optimal cutoff of 78.3% and specificity at the optimal cutoff of 44.3%) (Table 5).

## 4. Discussion

Our study first determined the features of circulating PGRN and EphA2 levels in Chinese patients with CAD and analyzed the association between PGRN and EphA2 levels and the atheroma burden in patients undergoing coronary angiography. Collectively, we reported three important findings [1]. Circulating EphA2 and PGRN levels are significantly higher in patients with ACS than in patients with SAP [2]. Levels of soluble N-terminal EphA2 fragments in blood were correlated with PGRN concentrations, the Gensini score, and the cardiac injury biomarkers in study subjects [3]. EphA2 and PGRN levels could statistically predict the presence of CTO in patients with CAD.

To our knowledge, increased PGRN levels might be novel biomarkers of chronic inflammatory diseases, such as chronic periodontitis and community-acquired pneumonia [20, 21]. Moreover, serum PGRN levels are reported to be associated with systemic inflammatory markers [22]. According to previous studies, mouse models deficient in PGRN and ApoE exhibit more severe atherosclerotic lesions than PGRN^(+/+)^ApoE^(-/-)^ mice [11]. However, the exact role of PGRN in atherosclerosis remains unclear [23].

In our study, patients with ACS had higher Gensini scores than non-ACS subjects (*p* < 0.001). We identified significant associations between PGRN levels, LVEF, CTO, and the levels of cardiac markers. Unlike the modest negative correlation between PGRN and high-density lipoprotein levels in Korean adults with CAD [23], we found that PGRN levels were not correlated with indices of glucose and lipid metabolism.

EphA2 is a member of the Eph receptor kinase family. Remarkably, the EphA2 gene is located in the region of human chromosome 1 (1p36) 16 associated with early myocardial infarction and the region of chromosome 4 associated with increased susceptibility to atherosclerosis (athsq1 locus) in mice [24, 25]. Circulating EphA2 is an effective biomarker to diagnose malignant tumors [26]. Recent studies have revealed the potential role of EphA2 in regulating plaque inflammation and progression [27]. Therefore, we detected levels of the soluble EphA2 fragment in the blood of patients with CAD. As expected, circulating EphA2 concentrations were positively correlated with the levels of cardiac injury markers (i.e., cTnT, NT-proBNP, hs-CRP, and CK series). Additionally, EphA2 levels were positively correlated with the Gensini score (*r* = 0.306, *p* < 0.001) but negatively correlated with the LVEF (*r* = −0.405, *p* < 0.001), suggesting that EphA2 may be a potential diagnostic biomarker for CAD.

Considering the potential significance of EphA2 and PGRN in inflammatory regulation and the progression of atherosclerosis formation, we applied a correlation analysis to assess the relationship between EphA2 and PGRN levels and found that EphA2 levels were moderately positively correlated with PGRN levels in all study subjects (*r* = 0.407, *p* < 0.001). In the prediction of ACS, we divided EphA2 levels into two groups based on a median of 116.32 pg/mL and included it in the multivariate logistic regression analysis with sex, levels of LDL, hemoglobinA1, and NT-proBNP, the Gensini score, and other factors. The OR value for EphA2 levels ≥ 116.32 pg/mL was 10.715 (95% CI: 3.259-35.227, *p* < 0.001).

Finally, we observed higher levels of circulating EphA2 and cardiac injury markers in patients with a greater atheroma burden than in those with a lower atheroma burden grouped by either the Gensini score or the existence of CTO. In addition, levels of EphA2 and PGRN significantly predicted CTO (AUC > 0.6, *p* < 0.05), suggesting the potential diagnostic efficiency of circulating EphA2 and PGRN levels in evaluating the atheroma burden in patients with CAD, which further confirms the roles of EphA2 and PGRN in the progression of atherosclerosis.

However, our study has some limitations. This study represented a small observational finding from a single center. Second, temporal changes (such as three months or 12 months) in EphA2 and PGRN levels were not considered before the study, which would make the conclusion more convincing. Finally, the present study illustrated but did not test the phenomenon of the synergistic mechanism of EphA2 and PGRN. More detailed study designs are needed to explore the specific molecular mechanism of the PGRN/EphA2 axis in CAD.

## 5. Conclusions

In summary, circulating EphA2 levels are significantly higher in patients with ACS. Our findings suggested the diagnostic value of circulating PGRN and EphA2 levels in predicting the atheroma burden in patients with CAD.

## Figures and Tables

**Table 1:** Baseline characteristics and biochemical index of the study participants.

Variables	Control (*n* = 42)	SAP (*n* = 75)	ACS (*n* = 84)	*p* value
Males, *n* (%)	30 (71.4)	54 (72.0)	69 (82.1)	0.094
Age (y) (mean ± SD)	62.3 ± 10.3	65.7 ± 9.1	66.2 ± 10.3	0.105
BMI (kg/m^2^)	24.6 ± 3.3	25.2 ± 2.6	24.5 ± 3.3	0.471
Cardiovascular risk factor				
Hypertension, *n* (%)	24 (57.1)	49 (65.3)	51 (60.7)	0.663
Diabetes mellitus, *n* (%)	10 (23.8)	28 (37.3)	35 (41.7)	0.141
Smoking, *n* (%)	14 (33.3)	34 (45.3)	34 (40.5)	0.447
Biochemical index				
eGFR (mL/min/1.73 m^2^)	90.0 (82.0, 95.0)	85.0 (70.0, 90.0)	83.0 (71.5,96.0)	0.041
Total cholesterol (mg/dL)	131.8 (109.0, 164.9)	118.3 (103.6, 151.9)^b^	152.7 (133.4, 185.6)^a^	<0.001
LDL (mg/dL)	56.1 (44.9, 87.4)^b^	48.9 (36.7, 75.8)^b^	85.4 (66.1, 116.4)^a^	<0.001
Hemoglobin A1c (%)	5.8 (5.5, 6.1)^b^	6.2 (5.7, 7.1)	6.4 (5.6, 7.9)^a^	0.023
NT-proBNP (pg/mL)	51.7 (32.9, 128.5)^b^	89.6 (44.9, 195.8)^b^	497.0 (137.8, 1050.0)^a^	<0.001
cTnT (ng/mL)	0.007 (0.005, 0.010)^b^	0.009 (0.006, 0.014)^b^	0.19 (0.04, 0.95)^a^	<0.001
CK-MB (U/L)	15.0 (13.0, 17.0)^b^	14.0 (12.0, 17.0)^b^	21.0 (14.5, 56.0)^a^	<0.001
hs-CRP (mg/L)	0.6 (0.3, 0.9)^b^	0.7 (0.4, 1.4)^b^	4.7 (1.0, 10.6)^a^	<0.001
PGRN (ng/mL)	41.2 (31.0, 50.1)	39.7 (31.4, 46.7)^b^	43.3 (37.5, 53.1)^a^	0.036
EphA2 (pg/mL)	39.0 (23.8, 109.6)^b^	38.8 (21.3, 173.9)^b^	284.5 (199.4, 437.6)^a^	<0.001
Gensini score	5.0 (1.5, 8.0)^c^	25.7 (16.5, 43.0)^b^	57.0 (38.0, 97.0)^a^	<0.001
LVEF (%)	66.0 (64.0, 67.0)^b^	64.5 (61.0, 67.0)^b^	58.0 (48.0, 62.0)^a^	<0.001
CTO	0 (0.0)	7 (9.5)	39 (46.4)	<0.001

Data are expressed as mean ± standard deviation or median (interquartile range) or *n* (%). ^a,b,c^Same letters indicate no statistical significance between groups based on Tukey's HSD post hoc test. ACS: acute coronary syndrome; SAP: stable angina pectoris; BMI: body mass index; eGFR: estimated glomerular filtration rate; LDL: low-density lipoprotein; NT-proBNP: N-terminal pro B-type natriuretic peptide; cTnT: cardiac troponin T; CK-MB: creatine kinase-MB; hs-CRP: high-sensitivity C-reactive protein; PGRN: progranulin; EphA2: Eph-receptor tyrosine kinase-type A2; LVEF: left ventricular ejection fraction; CTO: chronic total occlusion.

**Table 2:** Spearman's correlation of serum PGRN and EphA2 levels with risk factors and the Gensini score.

Variable	PGRN (ng/mL)		EphA2 (pg/mL)	
	*r*	*p* value	*r*	*p* value
Males, *n* (%)	-0.003	0.964	0.103	0.148
Age (y) (mean ± SD)	-0.018	0.800	0.221	0.002
BMI (kg/m^2^)	0.013	0.864	-0.147	0.054
Hypertension, *n* (%)	0.016	0.818	-0.021	0.772
Diabetes mellitus, *n* (%)	0.024	0.737	0.064	0.368
Smoking, *n* (%)	-0.051	0.474	-0.024	0.740
eGFR (mL/min/1.73 m^2^)	-0.075	0.303	-0.161	0.027
Total cholesterol (mg/dL)	0.078	0.278	0.186	0.008
LDL (mg/dL)	0.041	0.563	0.231	0.001
Hemoglobin A1c (%)	0.120	0.120	0.035	0.647
NT-proBNP (pg/mL)	0.185	0.009	0.389	<0.001
cTnT (ng/mL)	0.199	0.005	0.451	<0.001
CK-MB (U/L)	0.111	0.123	0.225	0.001
hs-CRP (mg/L)	0.182	0.010	0.278	<0.001
PGRN (ng/mL)	/	/	0.407	<0.001
EphA2 (pg/mL)	0.407	<0.001	/	/
Gensini score	0.099	0.170	0.306	<0.001
LVEF (%)	-0.215	0.003	-0.405	<0.001
CTO	0.147	0.041	0.330	<0.001

BMI: body mass index; eGFR: estimated glomerular filtration rate; LDL: low-density lipoprotein; NT-proBNP: N-terminal pro B-type natriuretic peptide; cTnT: cardiac troponin T; CK-MB: creatine kinase-MB; hs-CRP: hypersensitive C-reactive protein; PGRN: progranulin; EphA2: Eph-receptor tyrosine kinase-type A2; LVEF: left ventricular ejection fraction; CTO: chronic total occlusion.

**Table 3:** Univariate and multivariate logistic regression of risk factors of ACS.

Variable	Univariate			Multivariate		
	OR	95% CI	*p* value	OR	95% CI	*p* value
Males, *n* (%)	0.469	0.233~0.941	0.033	8.523	1.422~51.091	0.019
Age (y) (mean ± SD)	1.018	0.989~1.047	0.235			
BMI (kg/m^2^)	0.959	0.868~1.059	0.408			
Hypertension, *n* (%)	1.074	0.604~1.910	0.809			
Diabetes mellitus, *n* (%)	0.673	0.377~1.204	0.182			
Smoking, *n* (%)	1.023	0.578~1.810	0.938			
eGFR (mL/min/1.73 m^2^)	0.985	0.968~1.002	0.081			
Total cholesterol (mg/dL)	1.015	1.007~1.023	<0.001	0.972	0.943~1.001	0.058
LDL (mg/dL)	1.024	1.014~1.033	<0.001	1.050	1.014~1.087	0.006
Hemoglobin A1c (%)	1.511	1.162~1.963	0.002			
NT-proBNP (pg/mL)	1.002	1.001~1.003	<0.001	1.003	1.001~1.005	0.006
cTnT (ng/mL)	3.742*E* + 27	3.746*E* + 14 ~ 3.738*E* + 40	<0.001			
CK-MB (U/L)	1.080	1.037~1.125	<0.001			
hs-CRP (mg/L)	1.117	1.060~1.176	<0.001			
PGRN (ng/mL)	1.022	1.001~1.044	0.039			
EphA2 (pg/mL)	11.289	5.722~22.274	<0.001	10.715	3.259~35.227	<0.001
LVEF (%)	0.871	0.828~0.917	<0.001			
Gensini score	1.039	1.027~1.051	<0.001	1.031	1.014~1.048	<0.001

Forward Wald method was used in the multivariate logistic analysis. ACS: acute coronary syndrome; OR: relative risk; CI: confidence interval; BMI: body mass index; eGFR: estimated glomerular filtration rate; LDL: low-density lipoprotein; NT-proBNP: N-terminal pro B-type natriuretic peptide; cTnT: cardiac troponin T; CK-MB: creatine kinase MB; hs-CRP: hypersensitive C-reactive protein; PGRN: progranulin; EphA2: Eph-receptor tyrosine kinase-type A2; LVEF: left ventricular ejection fraction. EphA2 was divided into <116.32 pg/mL and ≥ 116.32 pg/mL according to the median.

**Table 4:** Differences between the lower and greater atheroma burden.

Parameter	Gensini < 41.0 (*n* = 80)	Gensini ≥ 41.0 (*n* = 79)	*p* value	Non-CTO (*n* = 113)	CTO (*n* = 46)	*p* value
Males, *n* (%)	60 (75.0)	62 (78.5)	0.708	83 (73.4)	39 (84.8)	0.150
Age (y) (mean ± SD)	65.3 ± 9.9	66.6 ± 9.6	0.510	66.3 ± 9.2	65.2 ± 11.1	0.764
BMI (kg/m^2^)	25.0 ± 3.0	24.7 ± 3.0	0.469	24.7 ± 2.8	25.1 ± 3.4	0.524
Hypertension, *n* (%)	55 (68.7)	45 (57.0)	0.141	69 (61.1)	31 (67.4)	0.476
Diabetes mellitus, *n* (%)	27 (33.7)	36 (45.6)	0.146	42 (37.2)	21 (45.6)	0.373
Smoking, *n* (%)	36 (45.0)	32 (40.5)	0.632	46 (40.7)	22 (47.8)	0.480
eGFR (mL/min/1.73m^2^)	83.0 (71.0, 91.0)	83.0 (71.0, 92.0)	0.710	85.0 (71.0, 91.0)	83.0 (71.0, 95.0)	0.188
Total cholesterol (mg/dL)	124.1 (107.1, 160.6)	145.4 (128.3, 181.9)	0.002	133.0 (106.9, 164.3)	152.7 (126.8, 214.6)	0.021
LDL (mg/dL)	55.3 (38.1, 93.6)	82.7 (62.8, 110.0)	<0.001	57.6 (39.8, 96.1)	84.1 (63.8, 124.9)	0.003
Hemoglobin A1c (%)	6.1 (5.6, 6.9)	6.6 (5.6, 7.7)	0.180	6.2 (5.6, 7.1)	6.4 (5.6, 7.7)	0.538
NT-proBNP (pg/mL)	119.7 (46.3, 311.7)	393.0 (132.0, 1089.0)	<0.001	140.0 (58.7, 373.6)	381.0 (112.0, 1040.0)	<0.001
cTnT (ng/mL)	0.01 (0.007, 0.045)	0.078 (0.018, 0.713)	<0.001	0.011 (0.008, 0.038)	0.064 (0.015, 0.633)	0.001
CK-MB (U/L)	15.0 (12.0, 18.0)	19.0 (13.0, 40.0)	0.004	14.0 (12.0, 19.0)	21.0 (14.0, 41.0)	0.009
hs-CRP (mg/L)	0.95 (0.45, 4.55)	4.80 (0.90, 14.85)	<0.001	1.00 (0.50, 4.55)	4.85 (0.90, 11.7)	0.003
PGRN (ng/mL)	40.1 (33.5, 50.6)	42.4 (37.4, 50.0)	0.208	39.4 (33.8, 46.7)	44.7 (39.7, 51.0)	0.052
EphA2 (pg/mL)	107.8 (27.0, 273.7)	243.8 (56.1, 368.8)	0.031	45.7 (23.1, 268.2)	298.0 (215.5, 379.2)	<0.001

Data are expressed as mean ± standard deviation or median (interquartile range) or *n* (%).CTO: chronic total occlusion. BMI: body mass index; eGFR: estimated glomerular filtration rate; LDL: low-density lipoprotein; NT-proBNP: N-terminal pro B-type natriuretic peptide; cTnT: cardiac troponin T; CK-MB:, creatine kinase-MB; hs-CRP: high-sensitivity C-reactive protein; PGRN: progranulin; EphA2:, Eph-receptor tyrosine kinase-type A2.

**Table 5:** Diagnostic efficiency of circulating EphA2 and PGRN levels to predict CTO.

Parameter	AUC	95% CI	Optimal cutoff	Sensitivity (%)	Specificity (%)	*p* value	Diagnose OR	Accuracy
PGRN (ng/mL)	0.604	0.508, 0.700	38.2	78.3	44.3	0.045	2.83	0.542
EphA2 (pg/mL)	0.686	0.600, 0.772	63.6	89.1	46.3	<0.001	7.23	0.592
Gensini score	0.911	0.864, 0.958	50.5	88.6	81.9	<0.001	38.84	0.845
NT-proBNP (pg/mL)	0.686	0.595, 0.777	209.4	73.9	61.5	<0.001	4.379	0.646
cTnT (ng/mL)	0.673	0.584, 0.763	0.018	71.7	56.9	0.001	3.806	0.608
CK-MB (U/L)	0.626	0.520, 0.731	20.5	54.5	75.2	0.016	3.778	0.699
hs-CRP (mg/L)	0.656	0.559, 0.752	4.65	58.7	70.6	0.003	3.402	0.671

PGRN: progranulin; EphA2: Eph-receptor tyrosine kinase-type A2; NT-proBNP: N-terminal pro B-type natriuretic peptide; cTnT: cardiac troponin T; CK-MB: creatine kinase-MB; hs-CRP: high-sensitivity C-reactive protein; CTO: chronic total occlusion.

## Data Availability

The datasets used and analyzed during the current study are available from the corresponding author on reasonable request.

## Abbreviations

ACS: Acute coronary syndrome
SAP: Stable angina pectoris
CAD: Coronary artery disease
CTO: Chronic total occlusion
ELISA: Enzyme-linked immunosorbent assay
EphA2: Eph-receptor tyrosine kinase-type A2
LVEF: Left ventricular ejection fraction
NT-proBNP: N-terminal pro B-type natriuretic peptide
PGRN: Progranulin
cTnT: Cardiac troponin T
hs-CRP: High-sensitive C-reactive protein
MCP-1: Monocyte chemotactic protein-1
IL-6: Interleukin-6
NSTEMI: Non-ST-segment elevation myocardial infarction
STEMI: ST-segment elevation myocardial infarction
UA: Unstable angina.
